# Dengue in Fiji: epidemiology of the 2014 DENV-3 outbreak

**DOI:** 10.5365/wpsar.2018.9.3.001

**Published:** 2019-05-15

**Authors:** Aneley Getahun, Anaseini Batikawai, Devina Nand, Sabiha Khan, Aalisha Sahukhan, Daniel Faktaufon

**Affiliations:** a School of Public Health and Primary Care, College of Medicine, Nursing and Health Sciences, Fiji National University, Suva, Fiji.; b Fiji Ministry of Health and Medical Services, Suva, Fiji.

## Abstract

**Introduction:**

Dengue virus serotype-3 caused a large community-level outbreak in Fiji in 2013 and 2014. We aimed to characterize the demographic features of affected individuals and to determine dengue mortality during the outbreak.

**Methods:**

All laboratory-confirmed dengue cases and deaths were included in this study. Incidence and mortality were calculated according to demographic variables.

**Results:**

A total of 5221 laboratory-confirmed cases of dengue were included in this analysis. The majority of patients were male (54.5%) and indigenous Fijians (iTaukei) (53.5%). The median age was 25 years old. The overall incidence was 603 per 100 000 population. The age-specific incidence was highest among people between 20 and 24 years of age (1057 per 100 000) for both sexes. The major urban and peri-urban areas of Suva and Rewa subdivisions reported the highest incidence of > 1000 cases per 100 000 population.

**​**

A total of 48 deaths were included in this analysis. The majority of dengue-related deaths occurred in males (62.5%) and in the iTaukei (60.4%) population. The median age at death was 35 years old. The overall dengue-related deaths was estimated to be 5.5 deaths per 100 000 population. Dengue mortality was higher for males (6.8 per 100 000) than females. The highest age- and sex-specific mortality of 18 per 100 000 population was among males aged 65 years and older.

**Discussion:**

Dengue morbidity and mortality were highest among males, indigenous people and residents of urban and peri-urban locations. Effective and integrated public health strategies are needed to ensure early detection and appropriate outbreak control measures.

## Introduction

Dengue is one of the most common vector-borne diseases of public health importance globally. The disease is endemic in more than 100 countries, (*1*) and it is estimated that 390 million dengue infections occur annually. (*2*) Dengue has emerged as a significant public health problem in Pacific island countries, including Fiji, causing large outbreaks in recent years. (*3*-*5*)

Dengue is endemic in Fiji, and its epidemiology has showed dynamic changes over the last four decades. (*6*-*8*) Dengue distribution is characterized by low endemic levels of transmission, usually dominated by a single serotype with cyclical patterns of outbreaks following introduction of a new serotype. (*4*, *7*) In non-outbreak years, the estimated incidence in Fiji ranged from 0.34 to 51.15 per 100 000 population. (*9*) Historical reports documented two nationwide outbreaks in 1971 and 1975, (*10*, *11*) after which there was no major outbreak for over a decade. Since 1988, outbreaks have occurred with increasing frequency, with six major outbreaks reported between 1998 and 2017. (*6*, *7*, *9*, *12*, *13*) Major outbreaks have occurred in a cyclical pattern of approximately every four to five years.

In 2013, dengue serotype-3 virus (DENV-3) re-emerged in the South Pacific after 18 years, causing concurrent outbreaks in several Pacific island countries. (*3*-*5*) Before then, DENV-3 had last circulated in Fiji in 1989 and 1990, causing a large community-level outbreak. At the end of 2013, dengue cases began to increase in Fiji, and an outbreak was declared that continued into 2014. During this outbreak, over 15 000 cases (1733 per 100 000) were reported nationwide with a record number of deaths. (*9*) We investigated demographic patterns of incidence and mortality during the 2014 outbreak period that could provide important information for the prediction and control of future outbreaks. We aimed to characterize dengue cases and to determine the magnitude of mortality in 2014.

## Methods

Dengue surveillance in Fiji includes notification of laboratory-confirmed and clinically suspected cases. Confirmatory testing is primarily performed at the national public health laboratory. The laboratory tests used in Fiji at the time of the outbreak were enzyme-linked immunosorbent assay and non-structural protein antigen. All confirmed cases are reported to the Fiji Centre for Communicable Disease Control (FCCDC). Laboratory surveillance data include demography (patient’s name, hospital number, age, sex, ethnicity, address, health facility), date of reporting, and test results. Information not routinely collected for surveillance purposes are date of onset, signs and symptoms, dengue type (dengue fever or dengue haemorrhagic fever), patient outcome (mortality) and socioeconomic data such as education level, occupation, and income. No established surveillance system for determining circulating serotypes exists; however, during outbreaks, representative blood samples are sent to overseas laboratories to identify the specific dengue virus causing the outbreak.

Health-care services in Fiji are provided by the Ministry of Health and Medical Services (MoHMS) which is divided into four divisions: Central, Northern, Western and Eastern. Each division is further divided into several subdivisions that have secondary-level (subdivisional) hospitals that receive referrals from community-level facilities. (*9*)

All health-care providers report suspected cases of dengue through the national notifiable disease surveillance schedule (NNDSS). The NNDSS staff monitors a comprehensive list of 46 diseases that medical officers are required to report weekly. The NNDSS only contains data on the number, age and sex of patients. All line lists of laboratory-confirmed cases are also submitted to the NNDSS during outbreaks.

Dengue deaths are reported using the standardized medical cause of death certificates (MCDC) that are completed by medical officers. Dengue must be explicitly stated on the MCDC to be coded as a dengue death. All deaths are registered into the patient information system (PATIS plus) electronic database using MCDC data and further coded according to the International Classification of Diseases, Tenth Revision (ICD 10) through an automated system called Iris (version 4.0). (*14*)

For our epidemiological review, all laboratory-confirmed dengue cases reported to FCCDC in 2014 were included. Duplicate entries of patients who were cases more than once within an incubation period were removed. Deaths attributed to dengue were obtained from PATIS plus. Three additional laboratory-confirmed dengue deaths in 2014 identified in another study (*15*) were added in the mortality analysis, even though they were not in the official record.

Data analysis was performed using Microsoft Office Excel 2010 and SPSS software version 24. Descriptive statistics were used for the demographic profile (sex, ethnicity and age) of laboratory-confirmed dengue cases and dengue-related deaths.

All descriptive analysis results were calculated as proportions and medians with interquartile ranges.

Overall and specific incidence and mortality, stratified by demographic variables, were computed using population projections provided by the Fiji Bureau of Statistics (FBOS) for 2014 (FBOS 2014, projected population, unpublished). Since FBOS data are not disaggregated for medical divisions, incidence and mortality by medical divisions and subdivisions were calculated using the 2014 population estimates from the MoHMS. (*9*) Incidence and mortality are expressed per 100 000 population. The geographic distribution of dengue patients was evaluated using the location of the treating health facilities since patients’ home addresses were not systematically recorded.

Ethical approval was given by the College Health and Research Ethics Committee, College of Medicine, Nursing and Health Science, Fiji National University and the National Health Research and Ethics Review Committee (2016.110.N.W.).

## Results

A total of 5249 laboratory-confirmed cases of dengue were reported in 2014 (FCCDC, unpublished). After excluding 28 duplicates, 5221 cases were included in this analysis. Most of the cases were male (54.5%), were iTaukei (indigenous Fijians) (53.5%) and were residents of the Central Division (65.5%) (**Table 1**). The median age (interquartile range [IQR]) was 25 years (16–35). The majority of cases (80%) were reported during the first quarter with the highest number of cases occurring during epidemiologic week 7 from 23 February to 1 March 2014 (614 cases).

**Table 1:** Demographic characteristics of laboratory-confirmed and dengue mortality cases

-	Confirmed dengue cases (*n* = 5 221) *n*(%)	Dengue deaths (*n* = 48) *n*(%)
**Sex**		
Male	2848 (54.5)	30 (62.5)
Female	2373 (45.5)	18 (37.5)
**Ethnicity**		
iTaukei	2794 (53.5)	22 (60.4)
Indian descent	2248 (43.1)	16 (33.3)
Others	179 (3.4)	3 (6.3)
**Age group**		
0–4	190 (3.6)	5 (10.4)
5–14	852 (16.3)	4 (8.3)
15–24	1464 (28.0)	9 (18.8)
25–34	1211 (23.2)	6 (12.5)
35–44	594 (11.4)	4 (8.3)
45–54	408 (7.8)	6 (12.5)
55–64	208 (4.0)	8 (16.7)
65+	123 (2.4)	6 (12.5)
Unknown	171 (3.3)	0
Median (IQR)	35 (18–57)	25 (16–35)
**Division**		
Central	3418 (65.5)	20 (40.7)
Western	1080 (20.7)	18 (37.5)
Northern	711 (13.6)	9 (18.8)
Eastern	12 (0.2)	1 (2.1)

IQR, interquartile range.

**Fig. 1:**
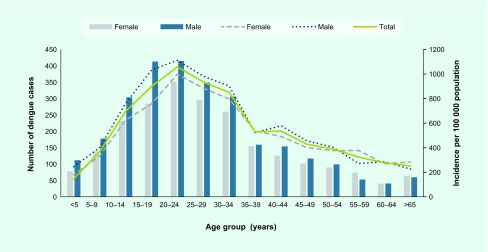
Age- and sex-specific incidence of laboratory-confirmed dengue in Fiji in 2014

**Fig. 2:**
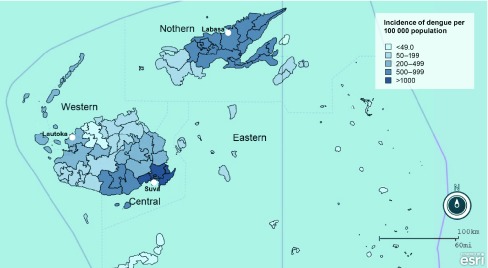
Dengue incidence by medical subdivision in Fiji in 2014

The overall incidence of laboratory-confirmed dengue was 603 cases per 100 000 population. The incidence was higher in males (674 per 100 000) compared with females (577 per 100 000). Age-specific incidence progressively rose after the age of 10 years and reached its highest among people between 20 and 24 years of age (1057 per 100 000) for both sexes, and it steadily declined in the age group 50 years and above (**Fig. 1**). Further analysis of the incidence by geographical location showed the highest burden in the Central Division (912 per 100 000) followed by the Northern and Western Divisions (542.8 per 100 000 and 278.6 per 100 000, respectively). The major urban and peri-urban subdivisions, within the Central Division (Suva and Rewa), reported the highest incidence of > 1000 per 100 000 population (**Fig. 2**).

**Fig. 3:**
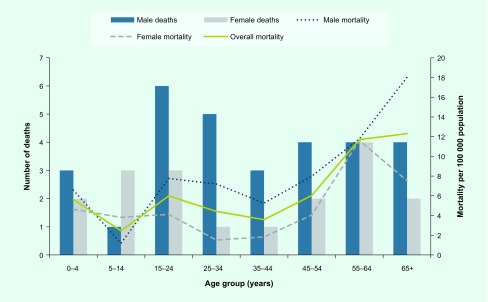
Dengue mortality in 2014 outbreak in Fiji, by age group and sex

A total of 45 deaths attributed to dengue were reported to MoHMS (PATIS plus, unpublished). Three additional dengue-related deaths were found during another study. (*15*) A total of 48 deaths were included in this analysis. The majority of reported deaths occurred in males (62.5%), iTaukei (60.4%) and residents of the Central Division (40.7%). The median IQR age at death was 35 years (18–57), and five (10%) deaths were among children under the age of 5 years. Overall mortality was estimated to be 5.5 deaths per 100 000 population. Dengue mortality was higher for males (6.8 per 100 000) compared to females (4.2 per 100 000). Mortality increased steadily with age: the highest mortality was among men aged more than 65 years (18 per 100 000) (**Fig. 3**). The lowest recorded mortality was for boys aged between 5 and 14 years. Those under 5 years of age had higher mortality (5.6 per 100 000) compared to children between 5 and 14 years of age (2.4 per 100 000). The Northern Division had the highest dengue mortality (6.9 per 100 000) followed by the Central (5.3 per 100 000) and Western Divisions (4.6 per 100 000). Two subdivisions in the North (Bua and Macuata) reported the highest mortality (12 per 100 000). Among notified deaths (*n* = 45), the underlying cause of death was reported as dengue fever in most (62.2%). The remaining 37.8% were reported as dengue haemorrhagic fever.

## Discussion

Dengue has emerged as a significant public health problem in Fiji and the South Pacific, causing large outbreaks in recent years. (*3*, *16*) A better understanding of the epidemiology of dengue is essential to appropriately allocate limited resources for dengue control and to better evaluate the impact of control activities. We conducted a retrospective review of dengue cases in Fiji during the 2014 DENV-3 outbreak to characterize the demographic features and to determine the magnitude of dengue-related mortality.

In this study, the median age of infection was 25 years, and age-specific incidence was highest among people between the ages of 15 and 34 years. The predominance among those in these age strata may be explained by their vulnerability to DENV-3, which last circulated in the country 24 years prior. Our findings are consistent with reports from previous outbreaks in Fiji that showed higher morbidity among adolescents and young adults, (*6*, *8*, *10*, *11*) which differs from patterns seen in hyperendemic countries in Asia where dengue is mainly reported among young children. (*17*) However, a shift in age groups has been demonstrated in some countries such as Singapore, Malaysia and Thailand, when dengue outbreaks occurred after the introduction of a new serotype mainly affecting the adult population. (*18*-*20*)

Males had a higher disease burden than females, as demonstrated by the increased absolute number and incidence among the male population. Previous outbreak and non-outbreak reports also demonstrated male preponderance in Fiji (*6*, *8*, *11*) and other countries. (*20*, *21*) We did not investigate the reason for the observed difference in incidence by sex, but possible reasons include increased risk of infection among men due to occupational exposure.

We report high dengue incidence in the main urban and peri-urban areas of Fiji. In the Central Division, Suva (the capital city) and Nausori, the adjacent peri-urban hub, reported the highest incidence with over 1000 cases per 100 000 population. Previous studies in Fiji reported a higher number of dengue cases and mosquito vectors in urban and peri-urban areas. (*8*, *22*, *23*) Increased reporting from urban areas could be due to greater availability of health services and access to testing. Urban and peri-urban areas in Fiji are characterized by expansive informal settlements with high population density and limited sanitation and public services. (*22*, *24*) The Suva–Nausori corridor in the Central Division has the largest concentration of informal settlements where access to clean water and sanitation may be an issue. (*25*) Globally, urban and peri-urban centres are identified as high-risk areas for dengue. (*2*) Increased risk of spread in these areas is attributed to population movement, travel, (*2*, *23*, *26*) overcrowding, increased vector breeding sites, (*26*) poor sanitation facilities and hygiene (*22*) and limited access to health care. (*19*) Dengue-prevention strategies in Fiji should consider the social determinants of health and include broader socioeconomic influences of better urban planning and improved sanitation to reduce the overall transmission risk factors.

During the 2014 outbreak, the majority of cases were reported in the first three months of the year. Previous outbreaks and surveillance reports showed similar seasonal patterns of dengue from November to April, coinciding with the warm and wet season. (*11*, *23*) This time period also overlaps with the cyclone season, when localized dengue outbreaks have been reported following heavy rain and flooding. (*27*) A previous study demonstrated a significant correlation between the incidence of dengue with high temperatures and increased rainfall in three study sites in Fiji. (*23*)

Mortality in the 2014 DENV-3 outbreak was higher than that of previously reported outbreaks in Fiji. The 1997–1998 DENV-2 outbreak (*7*) reported 13 deaths with an estimated mortality of 1.7 deaths per 100 000 population (based on the 1996 census). A review of the 1989–1990 DENV-1 outbreak (*6*) reported an estimated mortality of 2.1 deaths per 100 000 population (for the estimated population size in 1990). Our findings show higher mortality compared to other endemic and hyperendemic countries in South-East Asia and South America where dengue mortality during outbreak and non-outbreak years ranges from 0.1 to 0.5 per 100 000 population. (*28*, *29*) Literature suggests dengue deaths have increased over the last few decades in some countries and regions. (*28*-*30*) Increased mortality has also been attributed to greater health-seeking behaviour and increased sensitivity of surveillance for detecting dengue deaths. (*28*)

The higher mortality among males has been reported previously (*31*) and is thought to be due largely to differences in health-seeking behaviour. (*30*) In this study, mortality progressively increased after the age of 55 years, particularly for males. The high mortality among elderly patients has been attributed to decreased immunity, compromised organ function, underlying co-morbidities and prolonged hospitalizations, which increase the risk of hospital-acquired infection or secondary infection. (*32*, *33*) This trend is likely to continue as the population ages and the burden of noncommunicable diseases grows.

We found higher mortality in the Northern Division despite the relatively lower incidence. The Division is served by one divisional hospital (Labasa Hospital) and three subdivisional hospitals located in the main urban and peri-urban areas. While no studies have evaluated access to health services and quality of care in the Division, it has the highest poverty rate in the country and poor health indicators. (*25*) Health-seeking behaviours of the Northern Division population are likely limited by socioeconomic, geographic and infrastructure barriers, especially for rural communities. Labasa Hospital has few specialist doctors and a smaller intensive care unit compared to other divisional hospitals, which could impact the quality of care for critically ill patients, resulting in higher mortality. We cannot substantiate the quality of care and access to health services from the surveillance data. Further studies are warranted to determine the possible reasons of the increased mortality and to address health system-related issues.

In addition, the ethnic distribution of dengue mortality requires further investigation. Reports from a previous outbreak showed increased frequency of haemorrhagic manifestations in the iTaukei people; however, a larger proportion of deaths occurred in Fijians of Indian descent. (*6*) In contrast, in this outbreak, a large proportion of deaths occurred among the iTaukei people. A global review suggests that ethnic disparities in dengue severity remain unexplained. (*30*) One study shows that these differences may be largely due to socioeconomic factors that can be addressed by public health interventions. (*30*)

A review of the global literature on dengue mortality has highlighted underreporting and the difficulties associated with attributing dengue as a cause of death as factors challenging the understanding of mortality trends. In addition, heterogeneity in reporting of mortality and its predictors limits comparisons between studies. (*30*)

### Limitations

This study has several limitations. We limited our analysis to 2014 data to ensure a systematic line listing of cases. It is expected that given the broad clinical spectrum of dengue, many cases would not have been reported, particularly early in the outbreak (end of 2013). Between March and June 2014, only clinically suspected dengue patients were tested due to a shortage of dengue laboratory testing kits. Therefore, the numbers used in this study are likely to significantly underestimate the actual number of dengue cases and deaths that occurred in Fiji during this outbreak. In addition, incomplete case information (such as patient residential address, date of onset) further limits epidemiological analysis. Although residential address information was not available, geographical location of the treating health facility was considered appropriate as patients generally use the health facility closest to their residents. Health-care providers should systematically differentiate and specify the cause of dengue-related deaths, such as dengue shock and dengue haemorrhagic fever, for appropriate coding of underlying causes of death.

### Conclusions and recommendations

Vector-borne diseases remain a significant public health challenge in Pacific island countries and are expected to remain so due to a combination of environmental, climatic and socioeconomic factors. These factors increase the risk of transmission of dengue and emerging arboviral diseases such as chikungunya and Zika. (*3*) The high incidence and mortality described in this study indicate a need for continued surveillance of dengue in Fiji with regular assessments of its epidemiology to inform broad prevention strategies. We suggest that there is a need to integrate disease and vector surveillances to identify outbreaks earlier in high-risk areas. In addition, vector surveillance needs to be improved to provide real-time data on vector density in high-risk areas and to identify circulating serotypes before seasonal outbreaks occur. This will allow for early interventions to reduce breeding sites in targeted areas and inform risk communication strategies.

Early detection and prompt case management are crucial to reduce dengue mortality. We reported high mortality among males, indigenous people and residents of urban and peri-urban areas. This information needs to be incorporated into assessing high-risk patients and interventions for prevention. Further studies are required to identify specific risk factors for mortality among dengue patients in Fiji.

The 2014 DENV-3 outbreak in Fiji demonstrated the increasing risk of a large-scale community outbreak with increased mortality following introduction of a new dengue serotype.

Effective and integrated public health strategies are needed to ensure early detection and implement outbreak control measures.

## References

[1] Dengue and severe dengue. Geneva: World Health Organization; 2017 (https://www.who.int/mediacentre/factsheets/fs117/en/, accessed 5 January 2018).

[2] Bhatt S, Gething PW, Brady OJ, Messina JP, Farlow AW, Moyes CL, et al. The global distribution and burden of dengue. Nature. 2013 4 25;496(7446):504–7. 10.1038/nature12060 23563266 PMC3651993

[3] Roth A, Mercier A, Lepers C, Hoy D, Duituturaga S, Benyon E, et al. Concurrent outbreaks of dengue, chikungunya and Zika virus infections - an unprecedented epidemic wave of mosquito-borne viruses in the Pacific 2012-2014. Euro Surveill. 2014 10 16;19(41):20929. 10.2807/1560-7917.ES2014.19.41.20929 25345518

[4] Cao-Lormeau VM, Roche C, Musso D, Mallet HP, Dalipanda T, Dofai A, et al. Dengue virus type 3, South Pacific Islands, 2013. Emerg Infect Dis. 2014 6;20(6):1034–6. 10.3201/eid2006.131413 24856252 PMC4036764

[5] Nogareda F, Joshua C, Sio A, Shortus M, Dalipanda T, Durski K, et al. Ongoing outbreak of dengue serotype-3 in Solomon Islands, January to May 2013. West Pac Surveill Response. 2013 7 30;4(3):28–33. 10.5365/wpsar.2013.4.2.013 24319611 PMC3853998

[6] Fagbami AH, Mataika JU, Shrestha M, Gubler DJ. Dengue type 1 epidemic with haemorrhagic manifestations in Fiji, 1989-90. Bull World Health Organ. 1995;73(3):291–7. 7614660 PMC2486670

[7] Prakash G, Raju AK, Koroivueta J. DF/DHF and its control in Fiji. Dengue Bull. 2001;21:21–7. https://apps.who.int/iris/bitstream/handle/10665/163682/dbv25p21.pdf

[8] Erenavula JIJ, Ledua KS, Naicker P, Tukana VL, Kishore K. Dengue fever in Fiji: incidence from 2003–2009. Fiji Journal of Public Health. 2012;1(1):37–9. https://www.health.gov.fj/eJournal/index.php/2016/02/18/dengue-fever-in-fiji-incidence-from-2003-2009/

[9] Ministry of Health & Medical Services annual report 2014: Suva: Fiji Ministry of Health and Medical Services; 2014 (https://www.health.gov.fj/PDFs/Annual%20Report/Annual%20Report%202014.pdf, accessed 19 February 2016).

[10] Maguire T, Miles JAR, Macnamara FN, Wilkinson PJ, Austin FJ, Mataika JU. Mosquito-borne infections in Fiji. V. The 1971-73 dengue epidemic. J Hyg (Lond). 1974 10;73(2):263–70. 10.1017/S0022172400024116 4529580 PMC2130331

[11] Reed D, Maguire T, Mataika J. Type 1 dengue with hemorrhagic disease in Fiji: epidemiologic findings. Am J Trop Med Hyg. 1977 7;26(4):784–91. 10.4269/ajtmh.1977.26.784 889018

[12] Dengue fever situation in the Pacific island countries and territories, 30 September 2008. Noumea: Secretariat of the Pacific Community; 2008 (http://www.pphsn.net/ENGLISH/Publications/InformACTION/IA29/Selected_articles_per_disease-2.htm#dengue, accessed 19 December 2017).

[13] Pacific syndromic surveillance report, week 33, ending 20 August 2017. Manila: WHO Regional Office for the Western Pacific; 2017 (http://www.wpro.who.int/southpacific/programmes/communicable_diseases/disease_surveillance_response/PSS-20-August-2017/en/)

[14] International Statistical Classification of Diseases and Related Health Problems. 10th revision (ICD-10). Geneva: World Health Organization; 2010 (https://apps.who.int/classifications/icd10/browse/2016/en, accessed 3 March 2016).

[15] Getahun A, Batikawai A, Khan S, Nand D, Naidu R, Ram R, et al. Factors associated with dengue fatality in Fiji: a hospital-based case control study. Pac Health Dialog. 2019;21(3):139–47. 10.26635/phd.2019.603

[16] Dengue: guidelines for diagnosis, treatment, prevention and control. Geneva: World Health Organization; 2009 (https://www.who.int/tdr/publications/documents/dengue-diagnosis.pdf, accessed 3 February 2016). 23762963

[17] Bravo L, Roque VG, Brett J, Dizon R, L’Azou M. Epidemiology of dengue disease in the Philippines (2000-2011): a systematic literature review. PLoS Negl Trop Dis. 2014 11 6;8(11):e3027. 10.1371/journal.pntd.0003027 25375119 PMC4222740

[18] Limkittikul K, Brett J, L’Azou M. Epidemiological trends of dengue disease in Thailand (2000-2011): a systematic literature review. PLoS Negl Trop Dis. 2014 11 6;8(11):e3241. 10.1371/journal.pntd.0003241 25375766 PMC4222696

[19] Mohd-Zaki AH, Brett J, Ismail E, L’Azou M. Epidemiology of dengue disease in Malaysia (2000-2012): a systematic literature review. PLoS Negl Trop Dis. 2014 11 6;8(11):e3159. 10.1371/journal.pntd.0003159 25375211 PMC4222702

[20] Ler TS, Ang LW, Yap GS, Ng LC, Tai JC, James L, et al. Epidemiological characteristics of the 2005 and 2007 dengue epidemics in Singapore - similarities and distinctions. West Pac Surveill Response. 2011 5 20;2(2):24–9. 10.5365/wpsar.2010.1.1.011 23908885 PMC3730961

[21] Liew SM, Khoo EM, Ho BK, Lee YK, Omar M, Ayadurai V, et al. Dengue in Malaysia: factors associated with dengue mortality from a national registry. PLoS One. 2016 6 23;11(6):e0157631. 10.1371/journal.pone.0157631 27336440 PMC4919027

[22] Raju AK. Community mobilization in ***Aedes aegypti*** control programme by source reduction in peri-urban district of Lautoka, Viti Levu, Fiji Islands. New Delhi: WHO Regional Office for South-East Asia; 2003Available from: https://www.who.int/iris/handle/10665/163791

[23] Oli K, McNamara KE. Dengue and climate change: exploring the relationships and risks in Fiji. Fiji Journal of Public Health. 2015;4(1):1–7. https://www.health.gov.fj/pdfs/Fiji%20Journal%20of%20Public%20Health%20Vol4Issue1.pdf

[24] Greenwell J, McCool J, Kool J, Salusalu M. Typhoid fever: hurdles to adequate hand washing for disease prevention among the population of a peri-urban informal settlement in Fiji. West Pac Surveill Response. 2013 1 10;4(1):41–5. 10.5365/wpsar.2012.3.4.006 23908955 PMC3729110

[25] Children in Fiji: an atlas of social indicators. Suva: UNICEF in the Pacific; 2011 (https://www.unicef.org/pacificislands/Fiji_Equity_Atlas_Web_version.pdf, 23 February 2018).

[26] Singh N, Kiedrzynski T, Lepers C, Benyon EK. Dengue in the Pacific–an update of the current situation. Pac Health Dialog. 2005 9;12(2):111–9. 18181502

[27] Ministry of Health annual report 2012. Suva: Fiji Ministry of Health and Medical Services; 2012 (https://www.health.gov.fj/PDFs/Annual%20Report/Annual%20Report%202012.pdf, 15 July 2017).

[28] Wartel TA, Prayitno A, Hadinegoro SR, Capeding MR, Thisyakorn U, Tran NH, et al. Three decades of dengue surveillance in five highly endemic South East Asian countries. Asia Pac J Public Health. 2017 1;29(1):7–16. 10.1177/1010539516675701 28198645

[29] Paixão ES, Costa MC, Rodrigues LC, Rasella D, Cardim LL, Brasileiro AC, et al. Trends and factors associated with dengue mortality and fatality in Brazil. Rev Soc Bras Med Trop. 2015 Jul-Aug;48(4):399–405. 10.1590/0037-8682-0145-2015 26312928

[30] Carabali M, Hernandez LM, Arauz MJ, Villar LA, Ridde V. Why are people with dengue dying? A scoping review of determinants for dengue mortality. BMC Infect Dis. 2015 7 30;15(1):301. 10.1186/s12879-015-1058-x 26223700 PMC4520151

[31] Moraes GH, de Fátima Duarte E, Duarte EC. Determinants of mortality from severe dengue in Brazil: a population-based case-control study. Am J Trop Med Hyg. 2013 4;88(4):670–6. 10.4269/ajtmh.11-0774 23400577 PMC3617850

[32] Leo YS, Thein TL, Fisher DA, Low JG, Oh HM, Narayanan RL, et al. Confirmed adult dengue deaths in Singapore: 5-year multi-center retrospective study. BMC Infect Dis. 2011 5 12;11(1):123. 10.1186/1471-2334-11-123 21569427 PMC3112097

[33] Lee IK, Liu JW, Yang KD. Clinical and laboratory characteristics and risk factors for fatality in elderly patients with dengue hemorrhagic fever. Am J Trop Med Hyg. 2008 8;79(2):149–53. 10.4269/ajtmh.2008.79.149 18689614

